# Interventions for caries in primary teeth; mapping reported outcomes in clinical trials over the last 30 years

**DOI:** 10.1186/1745-6215-16-S1-P31

**Published:** 2015-05-29

**Authors:** Nicola P T Innes, Felicity Borrie, Neeraj Gugnani, Alex Keightley, Thomas J Lamont, Zoe Marshman, Ruth Santamaria, Falk Schwendicke

**Affiliations:** 1 University of Dundee, Dundee, DD1 4HR, UK; 2 DAV Centenary Dental College, Haryana, India; 3 University of Sheffield, Sheffield, UK; 4 University of Greifswald, Greifswald, Germany; 5 Charité – Universitätsmedizin Berlin, Germany

## Background

Dental caries (tooth decay) is the most prevalent disease in school age children and carries a burden of pain and infection. Clinical trials investigating interventions (fillings and other caries management techniques) to mitigate these have no agreed core outcome set making research design difficult and complicating evidence synthesis. The first step in addressing these issues is to map the current status.

## Research question

What outcomes have been measured in clinical trials investigating management of carious primary teeth over the past 30 years?

## Objectives

• Identify and categorise outcomes/outcome measures of all published randomised and controlled clinical trials investigating restoration of primary teeth in children, including pulp therapy;

• Assess quality of reporting; and

• Look for trends/gaps/patient reported/patient oriented outcomes.

### Inclusion

• Population: Individuals 6 months to 14 years (Human studies)

• Interventions: Management techniques for carious primary teeth and pulp therapy at tooth, patient, clinician or practice level.

• Comparisons/Control: No intervention/different interventions/Self

• Outcome: all outcomes

• Study design: randomised and controlled clinical trials

Search strategy for Cochrane Oral Health Group’s Trial register and Cochrane Central Register of Controlled Trials (CENTRAL): (((((("Dental Restoration, Permanent"[Mesh] OR "Dental Restoration, Temporary"[Mesh]) OR "Dental Cements"[Mesh]) OR "Pulp Capping and Pulpectomy Agents"[Mesh]) OR "Resins, Synthetic"[Mesh]) OR "Dental Pulp Capping"[Mesh]) OR "Pulpectomy"[Mesh]) OR "Pulpotomy"[Mesh] AND (Clinical Trial[ptyp] AND ("1983/11/01"[PDAT] : "2013/11/01"[PDAT]) AND "humans"[MeSH Terms] AND ("infant"[MeSH Terms] OR "child"[MeSH Terms] OR "adolescent"[MeSH Terms]))

## Data extraction and analysis

Titles/abstracts will be reviewed independently and in duplicate; obtain manuscripts for those meeting inclusion criteria, or where this is unclear; piloted proforma for data extraction, independently and in duplicate (3^rd^ person to resolve disagreement).

## Data management

Data will be entered into a bespoke Microsoft Access database; export to SPSS or Microsoft Excel.

## Data analysis

Outcomes/outcome measures listing; Independent categorisation by 3 researchers; consensus will be reached through discussion; Reporting quality will be measured according to Cochrane methodology; Outcome/outcome measure categories will be mapped against intervention type, year of publication, country of publication and author group; There will be discussion to interpret the findings, agree trends and look for gaps.

